# Insights into BRCA Cancer Predisposition from Integrated Germline and Somatic Analyses in 7632 Cancers

**DOI:** 10.1093/jncics/pkz028

**Published:** 2019-04-19

**Authors:** Shawn Yost, Elise Ruark, Ludmil B Alexandrov, Nazneen Rahman

**Affiliations:** 1 Division of Genetics and Epidemiology, Institute of Cancer Research, London, UK; 2 Department of Cellular and Molecular Medicine; 3 Department of Bioengineering, University of California, San Diego, La Jolla, CA; 4 Moores Cancer Center, University of California, San Diego, La Jolla, CA; 5 Cancer Genetics Unit, Royal Marsden NHS Foundation Trust, London, UK (NR)

## Abstract

**Background:**

It is often assumed any cancer in a germline *BRCA1* or *BRCA2* (collectively termed BRCA) mutation carrier was caused by that mutation. It is also often assumed the occurrence of breast or ovarian cancer in an individual with a variant of uncertain significance (VUS) suggests the VUS is pathogenic. These assumptions have profound management implications for cancer patients and healthy individuals.

**Methods:**

We compared the frequency of BRCA mutations, allele loss, and Signature 3 in 7632 individuals with 28 cancers and 1000 population controls. Because only increased frequency was the focus of the study, all statistical tests were one-sided.

**Results:**

Individuals with breast or ovarian cancer had increased germline BRCA pathogenic mutation frequencies compared to controls (*P* = 1.0x10^−10^ and *P* = 1.4x10^−34^, respectively). There was no increase in other cancer types. Wild-type allele loss and Signature 3 were statistically significantly higher in breast and ovarian cancers with BRCA mutations compared with other cancers with BRCA mutations (*P* = 5.1x10^−10^ and *P* = 3.7x10^−9^) and cancers without BRCA mutations (*P* = 2.8x10^−53^ and *P* = 1.0x10^−134^). There was no difference between non-breast and non-ovarian cancers with BRCA mutations and cancers without BRCA mutations. Allele loss and Signature 3 were statistically significantly higher in breast and ovarian cancers in individuals with BRCA pathogenic mutations compared to those with VUS (*P* = 3.8x10^−17^ and *P* = 1.6x10^−8^) or benign variants (*P* = 1.2x10^−28^ and *P* = 2.2x10^−10^). There was no difference between individuals with BRCA VUS and those with benign variants.

**Conclusions:**

These data show that non-breast and non-ovarian cancers in individuals with germline BRCA pathogenic mutations are often not causally related to the mutation and that BRCA VUS are highly unlikely to be pathogenic. These results should reduce inappropriate management of germline BRCA information.

*BRCA1* and *BRCA2* (collectively termed BRCA) are cancer predisposition genes (CPGs) that have been used in clinical practice for over 20 years (1). The genes were discovered through analyses of large families with breast and ovarian cancer, and their role in predisposing to these cancers is firmly established. Together, they contribute to approximately 15% of ovarian cancer and approximately 5% of breast cancer in most populations, with more in some isolated populations (1–3). The risks of breast and ovarian cancer associated with BRCA mutations is influenced by many factors, particularly family history. A prospective study of nearly 10000 BRCA mutation carriers gave cumulative breast cancer risks to 80 years of age 72% for *BRCA1* and 69% for *BRCA2*. For ovarian cancer, the risks were 44% for *BRCA1* and 17% for *BRCA2* (4). Studies have suggested much smaller increases in cancer risk and smaller contributions to cancer incidence for stomach, pancreas, uterine, cervix, and colon cancer for *BRCA1*, and prostate, pancreatic, gallbladder, stomach cancer, and melanoma for *BRCA2* (5–7).

Knowing if a cancer has been driven by a BRCA mutation is important for individualized cancer management, both for the current cancer and for the prevention and treatment of future cancers. This has become increasingly important with the advent of poly ADP-ribose polymerase inhibitor (PARP) inhibitors, which are used in the treatment of ovarian and breast cancers in individuals with BRCA mutations. There is also strong interest in the potential use of PARP inhibitors in individuals with BRCA mutations and other cancers (8).

It is commonly, but incorrectly, assumed any cancer occurring in an individual with a germline BRCA mutation has been caused by that mutation (9–13). This is despite consistent evidence that BRCA mutations predispose to a restricted set of cancers (5–7). Germline pathogenic BRCA mutations are present in about 1 in 250 individuals in most populations, with more in some less genetically heterogeneous populations (14,15). It is estimated about 1 in 2 people born after 1960 will get cancer (16). Thus, about 1 in 500 individuals is expected to have cancer and a germline BRCA mutation by chance alone. It is therefore paramount to ensure a BRCA mutation is causally related to a given cancer, prior to using treatments designed for BRCA-driven cancers.

Deciding which BRCA genetic variants are pathogenic is another common area of confusion leading to inappropriate clinical management (17–19). Studies have consistently shown the great majority of pathogenic BRCA mutations are protein-truncating variants (PTVs, also called loss-of-function variants) (15). Such mutations typically cause complete loss of function of the mutated allele because the truncation triggers nonsense-mediated RNA decay. The BRCA genes are large and highly polymorphic, and base substitutions that alter an amino acid nonsynonymous (missense) variants or do not alter an amino acid (synonymous variants) are collectively common (20). Only a small minority of nontruncating BRCA variants are pathogenic, and these are typically restricted to the ring finger and BRCT domains of BRCA1 and the DNA binding-domain of BRCA2 (15). Despite this large body of evidence, multiple studies have shown that up to 30% of rare, nontruncating BRCA variants are managed inappropriately as pathogenic mutations (17–19,21). This leads to inappropriate interventions in cancer patients and healthy individuals, and unnecessary costs to health services (15).

BRCA-driven cancers are characterized by distinctive somatic genetic features. *BRCA1* and *BRCA2* are tumor suppressor genes conforming to Knudson’s two-hit model. In cancers due to a germline BRCA mutation, the nonmutated (wild-type) copy of the gene is inactivated in the cancer, usually by allele loss. Less frequently, the wild-type BRCA gene is inactivated by somatic mutation (*BRCA1* and *BRCA2*) or promoter hypermethylation (*BRCA1*) (22). Most BRCA-driven cancers also have a mutational signature, known as Signature 3, characterized by substantial numbers of larger deletions with overlapping microhomology at breakpoint junctions (23).

Fast, affordable DNA sequencing is heralding an era of cancer diagnosis with upfront availability of germline and somatic sequencing data. This provides an opportunity to integrate these data to inform evaluations of the causal contribution of BRCA variants to a person’s cancer. Here, we have analyzed integrated germline and somatic BRCA data from 7723 individuals with 28 different cancers from The Cancer Genome Atlas (TCGA) to investigate these common assumptions about germline BRCA information.

## Methods

### TCGA Data Processing

We downloaded BAM files from 7723 individuals in the TCGA from CGHub, using genetorrent v3.8.3 (24,25); 7632 samples met our quality thresholds as detailed in the Supplementary Methods (available online).

We analyzed BAM files with OpEx v1.0 (26), using only OpEx high-quality calls in the analyses. There was an average of 21756 germline variants per TCGA exome, which is similar to the ICR1000 UK population exome series (average 21958 per exome), which we used as a comparison set (27). We used somatic variant and copy number calls from FireHose (https://gdac.broadinstitute.org). We used level 3 human 450k and 27k methylation ChIP data, which we analyzed using the methods in Nik-Zainal et al. (22) to identify extreme promoter hypermethylation of *BRCA1*. We used the methods in Alexandrov et al. (23) to determine the presence of mutational Signature 3.

### BRCA Analyses

We outputted all variants in the BRCA coding sequence or within 12 base pairs (bp) of the intron-exon boundaries. We stratified samples into three groups by BRCA germline variant status (Supplementary Table 1, available online). The pathogenic mutation group had a BRCA pathogenic mutation (n = 154). The rare variant group (n = 890) did not have a pathogenic mutation but had a BRCA rare variant, which we defined as a BRCA variant present at <0.1% variant frequency in the ExAC v0.3 non-TCGA dataset (20).The baseline cancers had neither a pathogenic mutation nor a rare variant, but they were heterozygous for a common nonsynonymous variant in *BRCA1* (c.2612C>T, n = 2167) or *BRCA2* (c.1114A>C, n = 2157). The baseline cancers were used to estimate the baseline rates of allele loss and Signature 3 in cancers (Supplementary Table 1, available online). A tumor sample was said to have loss of the *BRCA1* or *BRCA2* wild-type allele if the variant alternate allele frequency increased by more than 20% in the tumor (Tf) sample when compared to the matched germline (Gf) sample (Tf–Gf > 0.2). Further details are in the Supplementary Methods (available online).

### Statistical Analyses

We used the ICR1000 UK exome series to determine the population frequency of BRCA variants (27). For each cancer type, we calculated the probability of enrichment for germline *BRCA1* and *BRCA2* pathogenic mutations using a one-sided Fisher exact test using a Bonferroni corrected *P* value of 3.6 x 10^-4^ as the statistical significance threshold. We used a one-sided Fisher exact test to calculate the probability of cancers with pathogenic mutations or rare variants being statistically significantly enriched for loss of *BRCA1* or *BRCA2* wild-type allele when compared to baseline samples and to calculate the probability of observing more breast or ovarian cancers compared to other cancers with BRCA pathogenic mutations, loss of the wild-type allele, and Signature 3. We calculated the probability of cancers with pathogenic mutations or rare variants being statistically significantly enriched for normalized Signature 3 strength when compared to the baseline cancers using a one-sided Mann-Whitney U test. Further details are in the Supplementary Methods (available online).

## Results

### Datasets

We included 7632 individuals with 28 different cancer types in this study (Supplementary Table 1, available online). Germline variant calls were available for all 7632 samples and somatic variant calls for 6368 cancers. Somatic *BRCA1* methylation data were available for 7307 cancers, somatic copy number calls for 7227 cancers, and Signature 3 data for 4548 cancers. Tumor exome sequence data was used to investigate somatic BRCA allele loss in 3911 samples (Supplementary Figure 1, Supplementary Table 1, available online).

### Spectrum of Germline *BRCA1* and *BRCA2* Variation

**Figure 1.**
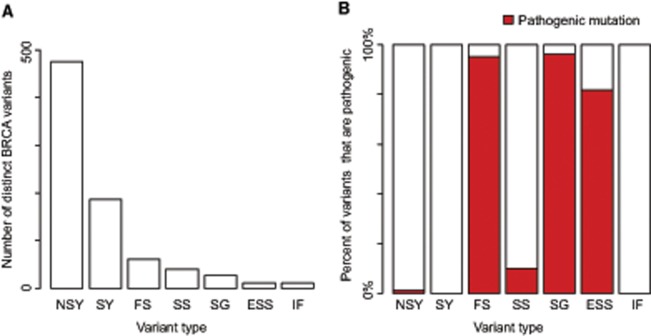
Spectrum of germline BRCA variants in 7632 individuals with cancer. **A)** Number and type of BRCA germline variants identified in 7632 individuals with 28 different cancer types. **B)** Percent of identified BRCA germline variants that are pathogenic mutations (**red**), by variant type. The majority of identified variants are nonsynonymous or synonymous variants, but <1% of these variants are pathogenic. By contrast, 94% of protein truncating variants (FS, SG, and ESS) are pathogenic. ESS = essential splice site; FS = frameshift; IF = in frame; NSY = nonsynonymous; SG = stop gain; SS = splice site; SY = synonymous.

We identified 813 distinct BRCA variants (Supplementary Table 2, available online): 100 protein-truncating variants (frameshift indels, stop-gain, or essential splice-site variants), 476 nonsynonymous base substitutions, 186 synonymous variants, 40 splice-site variants, and 11 in-frame indels (Figure 1A). We evaluated variant pathogenicity—that is, we determined which variants were proven to be associated with an increased risk of cancer. We referred to germline BRCA variants that unequivocally predispose to cancer as “pathogenic mutations,” in line with clinical convention.

All BRCA frameshift and stop-gain variants, except those after p.3325 in *BRCA2*, were designated pathogenic mutations; terminal *BRCA2* truncating variants do not confer the high cancer risks of other truncating mutations (28). Of the 11 essential splice-site variants, 9 were categorized as pathogenic mutations. *BRCA1* c.594-2A>C results in leaky splicing and is not associated with high cancer risks (29). *BRCA2* c.8954 + 5_8954 + 2delAACA leaves the splice junction intact. Thus, 94 of the 100 PTVs were designated pathogenic mutations. Of the remaining variants, 10 were designated pathogenic mutations because they either impact splicing to generate a truncated protein (n = 5) (30) or there was compelling genetic evidence of pathogenicity and, mechanistically, they impede BRCA1 function (n = 3) or BRCA2 function (n = 2). All were also independently classified as pathogenic by ClinVar (Supplementary Table 2, available online). There was no evidence that the remaining 703 variants were pathogenic. None fulfill the American College of Medical Genetics and Genomics (ACMG) guidelines for pathogenicity (31), and none were classed as pathogenic or likely pathogenic in ClinVar (Supplementary Table 2, available online).

In total, we identified 104 pathogenic mutations—46 in *BRCA1* and 58 in *BRCA2*—of which 99 are pathogenic because they lead to complete loss of function through generation of a truncated protein that likely causes nonsense-mediated RNA decay, and 5 are pathogenic through abrogation of BRCA function due to alteration of a critical amino acid (Supplementary Table 2, available online). The proportion of pathogenic mutations by variant class is shown in Figure 1B.

### Causal Contribution of Germline BRCA Pathogenic Mutations to Different Cancers

**Figure 2.**
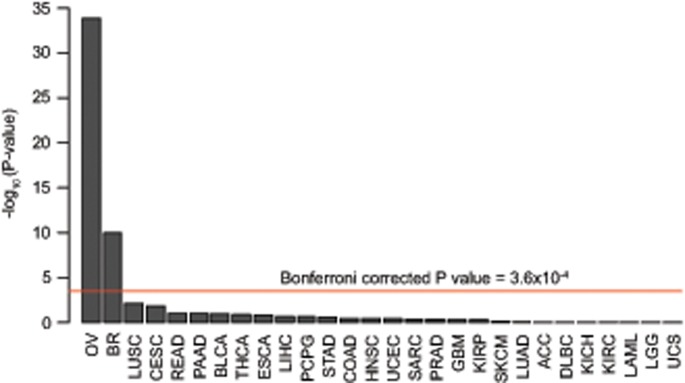
Individuals with breast or ovarian cancer have a statistically significant increase in germline BRCA pathogenic mutations. Bar plot of the probability, in negative log base 10 scale, of each of the 28 cancer types included in The Cancer Genome Atlas (TCGA) being enriched for germline BRCA pathogenic mutations, with the Bonferroni corrected *P* value of 0.01 (**red line**). Each cancer type was tested against the ICR1000 control set using a Fisher exact test. Ovarian serous cystadenocarcinoma (OV) and breast invasive carcinoma (BR) are the only cancer types from TCGA to be enriched for germline BRCA pathogenic mutations. Key to cancer code is given in Supplementary Table 3 (available online). *P* values are one-sided.

The 104 pathogenic BRCA mutations were present in 154 individuals (Supplementary Table 2, available online). The highest frequency of BRCA pathogenic mutations was 17.8% (66/370) in ovarian cancer followed by 5.0% (39/779) in breast cancer (Supplementary Table 3, available online). Of note, all ovarian cancers included in TCGA were high-grade serous ovarian cancers, and thus our results may not be representative of all ovarian cancer. The BRCA pathogenic mutation frequencies in ovarian and breast cancer are similar to other studies (2,3) and statistically significantly different to the ICR1000 UK population series in which there are four pathogenic BRCA mutations (Supplementary Table 4, available online) (*P* = 1.4x10^-34^ and *P* = 1.0x10^-10^ for ovarian cancer and breast cancer, respectively). The number of BRCA pathogenic mutations was not statistically significantly elevated for any other cancer type (Figure 2, Supplementary Table 3, available online).

**Figure 3.**
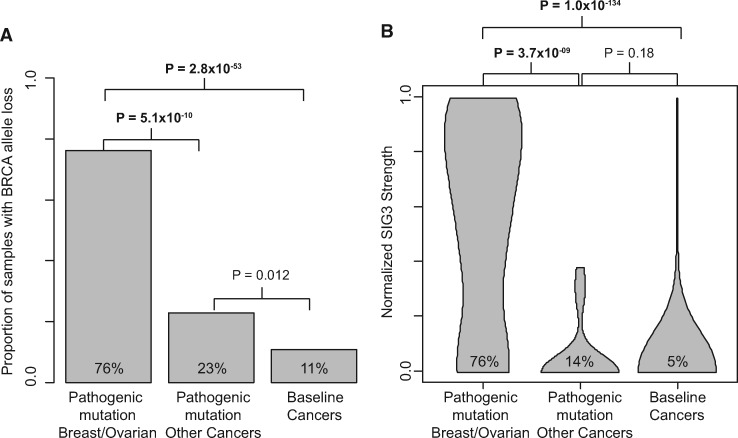
Comparison of BRCA allele loss and Signature 3 strength in individuals with BRCA pathogenic mutations by cancer type. **A)** Bar plot showing the proportion of samples with loss of the BRCA wild-type allele in individuals with breast or ovarian cancer and a BRCA pathogenic mutation Breast and Ovarian vs individuals with non-breast and non-ovarian cancer and BRCA pathogenic mutation (Other Cancers) vs cancers in individuals without BRCA pathogenic mutations or rare variants (Baseline Cancers). **B)** Violin plot of the normalized Signature 3 strength in the same groups. The width of the bar corresponds to the concentration of samples with a given normalized Signature 3 strength. Pairwise comparison *P* values, from a Fisher exact test, are shown. Statistically significant *P* values are in bold. *P* values are one-sided.

To further explore the contribution of germline BRCA pathogenic mutations to cancers, we investigated BRCA allele loss and Signature 3 presence. Tumor data was available from 153 of 154 individuals with pathogenic mutations, of which 91 showed evidence of loss of the wild-type allele. We determined the baseline rates of allele loss using data from 2923 baseline cancers without pathogenic or rare BRCA variants that were heterozygous for a common BRCA variant that allowed determination of allele loss; 76% (80/105) of breast and ovarian cancers from individuals with a germline pathogenic mutation exhibited loss of the wild-type allele compared with 23% (11/48) of other cancer types with a germline pathogenic mutation (*P* = 5.1x10^-10^) and 11% (312/2923) of baseline cancers (*P* = 2.8x10^-53^) (Figure 3A;Supplementary Table 1, available online). By contrast, there was only a marginal difference in BRCA allele loss between non-breast and non-ovarian cancers with germline pathogenic mutations and the baseline cancers (*P* = .012) (Figure 3A;Supplementary Table 1, available online).

We next evaluated the Signature 3 data, which was available for 111 cancers from individuals with BRCA pathogenic mutations and 2568 baseline cancers. Of the cancers with pathogenic mutations, 76% (63/83) of breast and ovarian cancers had Signature 3 compared with 14% (4/28) of other cancer types (*P* = 3.7x10^-9^) and 5% (130/2568) of baseline cancers (*P* = 1.0x10^-134^) (Figure 3B;Supplementary Table 1, available online). There was no difference in Signature 3 between the non-breast and non-ovarian cancers with pathogenic mutations and the baseline cancers (*P* = .18) (Figure 3B).

Together these data confirm genetic evidence that most ovarian and breast cancers in individuals with germline BRCA pathogenic mutations are causally related to that germline mutation. However, for many other cancers, there is likely no causal relationship between the cancer and the germline BRCA mutation.

### Causal Contribution of Rare BRCA Variants to Breast and Ovarian Cancers

**Figure 4.**
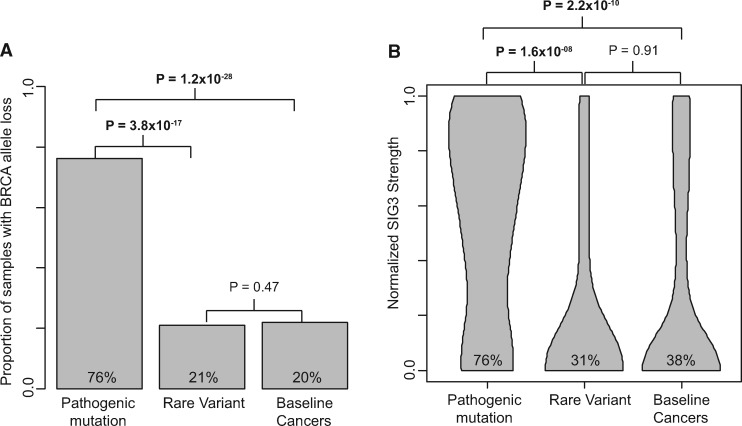
Comparison of BRCA allele loss and Signature 3 strength in breast and ovarian cancer, by germline BRCA variant type. **A)** Bar plot showing the proportion of samples with loss of the BRCA wild-type allele in breast and ovarian cancers in individuals with BRCA pathogenic mutations vs individuals with rare variants vs individuals without pathogenic mutations or rare variants (Baseline Cancers). **B)** Violin plot of the normalized Signature 3 strength in breast and ovarian cancers in the same groups. The width of the bar corresponds to the concentration of samples with a given normalized Signature 3 strength. Pairwise comparison *P* values, from a Fisher exact test, are shown. Statistically significant *P* values are in bold. *P* values are one-sided.

We next investigated variant pathogenicity in individuals with breast or ovarian cancer and a germline BRCA variant. Loss of the wild-type allele was observed in 76% (80/105) breast and ovarian cancers with pathogenic mutations compared with 21% (25/119) breast and ovarian cancers with rare variants (*P* = 3.8x10^-17^) and 20% (124/613) baseline breast and ovarian cancers without pathogenic or rare variants (*P* = 1.2x10^-28^). By contrast, there was no difference between the breast and ovarian cancers with rare variants and the baseline cancers (*P* = .47) (Figure 4A).

Similarly, 76% (63/83) breast or ovarian cancers with germline pathogenic mutations and Signature data had Signature 3 compared with 31% (18/59) breast or ovarian cancers with germline rare variants (*P* = 1.6x10^-8^) and 38% (113/301) baseline breast and ovarian cancers (*P* = 2.2x10^-10^). There was no difference in Signature 3 between the breast and ovarian cancers with rare BRCA variants and the baseline (*P* = .91) (Figure 4B).

Finally, we evaluated how many breast and ovarian cancers with Signature 3 were caused by two somatic BRCA events using 187 breast and ovarian samples with Signature 3 for which somatic variant calls, methylation data, and copy number calls were available. No (0/60) cancer with a germline pathogenic mutation had two somatic BRCA events compared with 11% (2/18) of cancers with a germline rare variant (*P* = .051) and 21% (23/109) of baseline cancers (*P* = 2.5x10^-5^) (Supplementary Table 5, available online). There was no difference between the number of cancers with a germline rare BRCA variant and two somatic BRCA events and the baseline cancers (*P* = .52).

These data demonstrate that rare nontruncating germline BRCA variants, which are typically reported as variants of uncertain significance, are very unlikely to predispose to cancer.

## Discussion

Precision oncology aspires to use genetic information to select the best treatments for cancer patients. To date, most precision oncology has focused on somatic mutations. However, germline genetic mutations also have therapeutic utility and are increasingly available at cancer diagnosis.

It is known that many somatic mutations are “passengers” rather than “drivers” and are not causally related to the cancer in which they occur (32). A major challenge for precision oncology has been identifying the somatic mutations that are drivers in an individual cancer (32). This same challenge applies at the germline level, but this has not been well appreciated. Indeed, it is often assumed a cancer occurring in an individual with a germline CPG mutation must be causally related to that mutation. This erroneous assumption occurs despite extensive evidence showing all CPGs are associated with a limited set of cancers. There is no pan-cancer CPG.

Establishing the spectrum of cancers associated with a CPG has traditionally been performed through genetic epidemiological studies of relatives of mutation carriers to deduce which cancers occur more frequently than expected by chance (5–7). A complementary approach is to perform germline CPG sequencing in cancer patients to see if the mutation frequency is greater than in the general population. Here, we have undertaken this approach for *BRCA1* and *BRCA2* in 28 cancer types. There are limitations to our analyses; in particular, although the total number of cancers is reasonably large, some individual cancers are not well represented. Also, some of our analytical methods have been superseded since we performed the analyses, for example, for determining copy number or methylation. However, none of these alternative methods would be expected to have a differential impact in the different subgroups we have evaluated. Thus, although it would be of potential interest to apply newer approaches, it would not be expected to alter our findings.

As expected, the BRCA pathogenic mutation frequencies in breast and ovarian cancer were statistically significantly higher than the population frequencies. However, there was no difference for any other TCGA cancer, and the BRCA pathogenic mutation frequency in non-breast and non-ovarian cancers (49/6483, 0.76%) is similar to the population frequency (4/993, 0.40%; *P* = .15). Moreover, the majority of breast and ovarian cancers occurring in individuals with a germline BRCA mutation exhibit loss of the wild-type BRCA allele and Signature 3. By contrast, this triad of features was very rare in non-breast and non-ovarian cancers.

Taken together, these data suggest germline BRCA pathogenic mutations in patients with cancers other than breast and ovarian cancer are often incidental findings. They reflect the population frequency of BRCA mutations rather than a causal relationship to the patient’s cancer. For certain non-breast and non-ovarian cancers, such as pancreatic cancer (2/107, 1.9%) and prostate cancer (2/345, 0.6%), studies show the likelihood of a causal relationship is higher, particularly in certain patient subgroups (5–7,33, 34). Nevertheless, given the penetrance and contribution of BRCA mutations in non-breast and non-ovarian cancers are low, clarification as to whether the germline mutation is a driver or an incidental finding would be prudent, prior to instituting BRCA-specific management.

Identification of an incidental BRCA mutation in cancer patients still has clinical utility. It will likely not influence treatment of the incidental cancer, but it will have risk implications for BRCA-related cancers, both for the cancer patient and relatives. *BRCA1* and *BRCA2* are included in the genes recommended by ACMG to be returned if an incidental mutation is identified (35). The frameworks for return and management of incidental BRCA mutations are equally applicable to cancer and noncancer patients. However, increased awareness of the possibility and implications of finding an incidental BRCA mutation during genetic testing in cancer patients is urgently required. The underlying principles and management frameworks are also applicable to other cancer predisposition genes, particularly the more common, lower penetrance CPGs such as *ATM* and *CHEK2*, which will often be incidental findings (36). A recent study estimated 8% of cancers occur in individuals with a CPG pathogenic mutation, demonstrating this is an important issue (37).

Management of individuals with rare, nontruncating BRCA variants is another challenging area causing confusion and clinical harms (17). Such variants are typically reported as variants of uncertain significance (VUS). The BRCA VUS rate of test providers is extremely variable but can be as high as 20% (17–19,21). It is recommended individuals with a BRCA VUS are managed in the same way as individuals with a benign variant, because the available evidence strongly indicates the vast majority do not confer increased risks of cancer (15). However, currently, appreciable numbers of VUSs are being managed as pathogenic mutations, with some studies showing that 30%–40% of healthy women with a BRCA VUS are having prophylactic mastectomies and oophorectomies (17–19).

The data presented here clearly show breast and ovarian cancers in individuals with a BRCA VUS are similar to non-BRCA breast and ovarian cancers. The rates of allele loss and Signature 3 are not elevated and are statistically significantly lower than cancers occurring in individuals with pathogenic BRCA mutations. Hence, the great majority of breast and ovarian cancers in individuals with a BRCA VUS are not causally related to the VUS and have arisen coincidentally. These data provide strong experimental support for the recommendation for BRCA VUS to be managed as benign variants (35). We hope our data will help patients and clinicians have more confidence in the recommendations so the unacceptably high rate of inappropriate interventions in individuals with BRCA VUS can be reduced (18–21).

Our data also suggest the integration of somatic genetic information may be a useful adjunct to variant interpretation, consistent with other studies (38). However, although the absence of loss of the wild-type allele and Signature 3 supports a rare nontruncating BRCA variant not being pathogenic, the opposite cannot be assumed. That is, the presence of allele loss and/or Signature 3 should not be taken as evidence that a rare nontruncating variant is pathogenic, as both occur at appreciable levels in cancers not driven by BRCA mutations. It should also be noted that although the majority of breast and ovarian cancers driven by pathogenic germline truncating BRCA mutations will exhibit loss of the wild-type allele and/or Signature 3, about one-quarter of such cancers in this study did not have one or other of these features, although only three cancers had neither.

Finally, our study adds to publications demonstrating the utility of integrating germline and somatic genetic data to inform the role of cancer predisposition genes in driving oncogenesis (37,38). As germline and somatic genetic information becomes routinely available at cancer diagnosis, we need to develop the analytical pipelines required to leverage these data to implement precision oncology correctly, consistently, and robustly.

## Funding

Royal Marsden/ICR NIHR Specialist Biomedical Research Centre for Cancer.

## Notes

Affiliations of authors: Division of Genetics and Epidemiology, Institute of Cancer Research, London, UK (SY, ER, NR); Department of Cellular and Molecular Medicine (LBA) and Department of Bioengineering, University of California, San Diego, La Jolla, CA (LBA); Moores Cancer Center, University of California, San Diego, La Jolla, CA (LBA); Cancer Genetics Unit, Royal Marsden NHS Foundation Trust, London, UK (NR). NR is a nonexecutive director of AstraZeneca. ER is now a product manager for Foresite Capital. SY is now a senior computational biologist at EngineBio.

SY, ER, and NR designed the experiment and undertook the analyses. LA performed and provided the Signature data. SY and NR wrote the paper, with input from ER and LA.

We are grateful to ICR Scientific Computing for assistance in accessing the TCGA datasets used in this study and to Ann Strydom for assistance in preparing the manuscript.

## Supplementary Material

Supplementary_Table_pkz028
