# Supplementary material for: Insights into BRCA Cancer Predisposition from Integrated Germline and Somatic Analyses in 7632 Cancers
Source: JNCI Cancer Spectr. 2019 Apr 19;3(2):pkz028. doi: 10.1093/jncics/pkz028 (PMC6649772; doi:10.1093/jncics/pkz028)
Supplement: Supplementary_Table_pkz028 [file supplementary_table_pkz028.zip › 20181107 BRCATCGA Supplementary Material.pdf]

Supplementary Material

Insights into BRCA cancer predisposition from integrated germline and somatic analyses in 7632 cancers

Shawn Yost[1], Elise Ruark[1], Ludmil B. Alexandrov[2,3,4], Nazneen Rahman[1,5]

Supplementary Methods

Figure S1: Summary of TCGA data used

Table S1: Summary of data available per sample

Table S2: Summary of 813 BRCA variants identified in the 7632 individuals with cancer

Table S3: Number of samples of the 28 cancer types investigated and the number with germline BRCA pathogenic mutations

Table S4: Summary of BRCA variants identified in 1000 UK population controls

Table S5: Summary of germline and somatic BRCA data in 187 breast/ovarian cancers with Signature 3

Corresponding Author: Nazneen Rahman email: rahmanlab@icr.ac.uk

Supplementary Methods

TCGA data download

We downloaded BAM files from CGHub,(25).using genetorrent v3.8.3. We downloaded germline Illumina exome sequencing data from 7,723 individuals in the TCGA that were available on 13/05/2014 for which the sample type was "Blood Derived normal", "Bone Marrow Normal", or "Solid Tissue Normal". We downloaded somatic exome sequencing data from the matched tumor sample using the sample type "Additional – New Primary", "Primary Blood Derived Cancer – Bone Marrow", "Primary Blood Derived Cancer – Peripheral Blood", or "Primary Solid Tumor". If there was more than one associated BAM file for a germline or tumor sample we selected the BAM file with the largest file size.

Exome processing and variant calling

We analysed all the BAM files with OpEx v1.0 (26). to provide consistent annotation of variants with the HGVS-compliant CSN (Clinical Sequencing Notation) standard v1.0 using NM_007294.3 for *BRCA1* and NM_000059.3 for *BRCA2*. We only used high quality calls, as defined by OpEx, in the analyses. Of note, the data was not of sufficient quality to call germline exon deletions or duplications which account for ~10% of pathogenic BRCA mutations (15).

Sample exclusions

We excluded a sample from the analyses if it did not meet all of the following criteria: heterozygosity (h) ratio $0.51 \leq h \geq 0.69$, number of indels (i) $347 \leq i \geq 1210$, transition to transversion (Ti/Tv) ratio $2.79 \leq Ti/Tv \geq 3.43$, ≤25% called variants were "low" quality variants, and >50% coding bases were covered by at least 30 reads. The minimum and maximum cutoff values for h, i, and Ti/Tv were chosen because they were +/- 4 standard deviations from the mean. 91 of the 7,723 germline exomes failed to meet these criteria. The remaining 7,632 samples were used in the analyses (Supplementary Table 1). There was an average of 21,756 germline variants per TCGA exome which is similar to the ICR1000 UK exome series (average 21,958 per exome), which was used as a comparison set for some analyses (27).

Somatic variant calls

We used the somatic variant calls from FireHose (https://gdac.broadinstitute.org) which we accessed using firehose_get. We downloaded all level 3 MAF files available on 21/08/2015. These were converted into VCF files using an in-house script (available on request). The VCF files were then annotated with CAVA v1.2 to ensure consistent germline and somatic variant annotations.

Somatic copy number calls

We used copy number calls from FireHose. We downloaded all level 4 Gistic2 files available on 28/01/2016 and extracted the *BRCA1* and *BRCA2* copy number status for each available sample from the "all_data_by_genes.txt" file.

BRCA1 methylation

We downloaded level 3 human 450k and 27k methylation ChIP data from the TCGA bulk data website (https://tcga-data.nci.nih.gov/tcga/findArchives.htm) on 27/07/2015. We used the methods in Nik-Zainal et al. to identify extreme promoter hypermethylation of *BRCA1* (22).In summary we extracted the methylation probes 5kb upstream and 1kb downstream from the *BRCA1* transcription start site. We defined a probe as being hypermethylated if its beta value was higher than the outlier threshold for that probe. (22). A sample was defined as having *BRCA1* promoter hypermethylation if 5 or more probes were hypermethylated.

Signature 3 analysis

The presence of mutational Signature 3 was determined using the methods described in Alexandrov et al (23). Samples with fewer than 50 somatic mutations were excluded from Signature analyses. We normalized the Signature 3 strength for each sample by dividing the Signature 3 strength by the sum of all Signature strengths in the sample. Tumors with Signature 3 strength >0 were considered to have Signature 3 ('Yes' in Supplementary Table 1). Tumors with Signature 3 strength =0 were considered to not have Signature 3 ('No' in Supplementary Table 1)

Stratification by BRCA germline status

We outputted all variants in the BRCA coding sequence or within 12bp of the intron-exon boundaries. We classified variants into one of seven groups: 1) Frameshift insertions/deletions (FS), 2) stop-gain (also called nonsense) variants (SG) 3) essential splice-site variants (ESS). Collectively the frameshift, stop-gain and essential splice-site variants are termed protein truncating variants (PTVs), because these variant classes are predicted to lead to premature protein truncation. 4) Nonsynonymous (also called missense) (NSY), 5) synonymous (SY), 6), splice-site (SS - encompassing CAVA groups EE, INT, SS and SS5) and 7) inframe deletions/insertions (IF).

We stratified the samples into three groups by BRCA germline variant status for some analyses (Supplementary Table 1). The 'Pathogenic Mutation' group had a BRCA pathogenic mutation (n=154). The 'Rare Variant' group (n=890) did not have a pathogenic mutation but had a BRCA 'rare variant' which we defined as a BRCA variant present at <0.1% variant frequency in the ExAC v0.3 non-TCGA dataset (20).The 'Baseline cancers' had neither a pathogenic mutation nor a rare variant, but were heterozygous for a common nonsynonymous variant in *BRCA1* (c.2612C>T,

n=2,167) or *BRCA2* (c.1114A>C, n=2,157). The Baseline cancers were used to estimate the baseline rates of allele loss and Signature 3 in cancers (Supplementary Table 1).

Loss of *BRCA1* or *BRCA2* wildtype allele

A tumor sample was said to have loss of the *BRCA1* or *BRCA2* wildtype allele if the variant alternate allele frequency increased by more than 20% in the tumor (Tf) sample when compared to the matched germline (Gf) sample (Tf - Gf > 0.2). An increase of 20% was used because it is greater than 2 standard deviations from the average germline allele frequency in *BRCA1* and *BRCA2*. We used a pathogenic mutation to evaluate allele loss if available otherwise we used a rare variant or common variant as defined above. For samples with >1 rare variant we selected the variant with the lowest frequency in ExAC to evaluate allele loss. For the 1,113 samples that were heterozygous for both the *BRCA1* and *BRCA2* common nonsynonymous variants we randomly selected one of the two common variants to evaluate allele loss.

Statistical Analyses

We used the ICR1000 UK exome series to determine the population frequency of BRCA variants (27). For each cancer type we calculated the probability of significant enrichment for germline *BRCA1* and *BRCA2* pathogenic mutations using a one-sided Fisher's Exact test, which we implemented with the fisher.test function in R and setting alternative equal to greater. To correct for multiple testing we used a Bonferroni corrected P-value of 3.6×10^{-4} as the significance threshold. We used the same one-sided Fisher's Exact test to calculate the probability of cancers with pathogenic mutations or rare variants being significantly enriched for loss of *BRCA1* or *BRCA2* wildtype allele when compared to baseline samples. The same one-sided Fisher's Exact test was used to calculate the probability of observing more breast/ovarian cancers compared to other cancers with BRCA pathogenic mutations, loss of the wild-type allele and Signature 3. We calculated the probability of cancers with pathogenic mutations or rare variants being significantly enriched for normalized Signature 3 strength when compared to the baseline cancers using a one-sided Mann-Whitney *U* test; which we implemented with the wilcox.test function in R, setting alternative equal to greater and paired equal to FALSE.

Figure S1. Summary of the TCGA datasets used

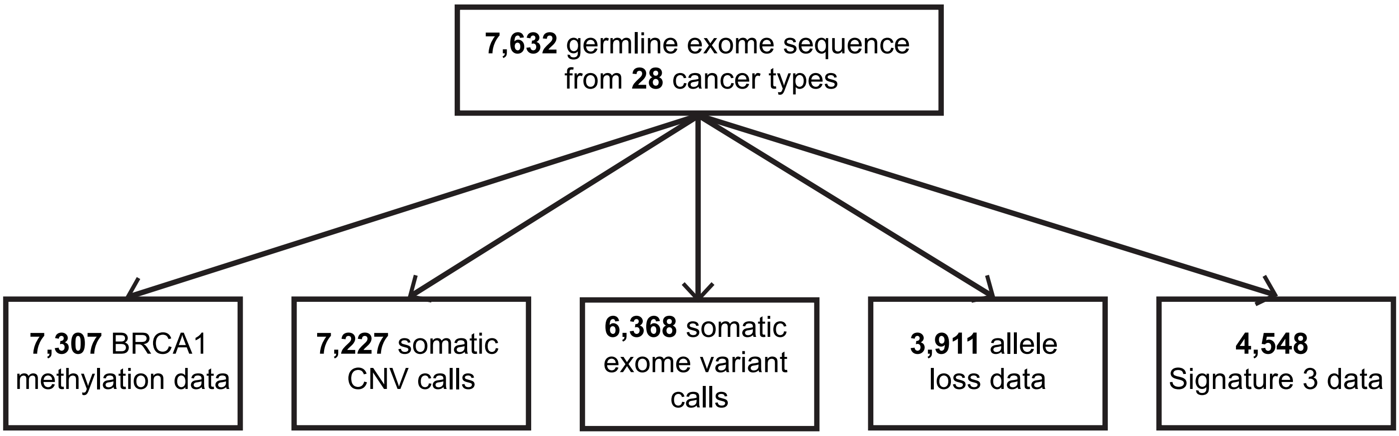

The number of samples available for each data types is given. Full, per sample details are provided in Table S1.

Table S1. Summary of data available per sample

Description of column headings are given below:

ID = TCGA Barcode; Cancer = abbreviation of the primary cancer diagnosis; AgeAtDx = Age at diagnosis; Gender = sample's gender; MethylationData = 'Yes'/'No' methylation data was available on 27/07/2015; SomaticCNVData = 'Yes'/'No' Gistic2 copy number data was available on 28/01/2016; SomaticVariantCalls = 'Yes'/'No' somatic variant call data from FireHose was available on 21/08/2015; AlleleLoss = 'Yes'/'No' the sample showed evidence for loss of BRCA1 or BRCA2 wildtype allele (see methods for details) or '.' the sample could not be assessed for loss of BRCA wildtype allele; Signature 3 presence = 'Yes'/'No' the sample has a Signature 3 strength > 0 or '.' Signature 3 strength could not be assessed for that sample; Signature3 strength = normalized Signature 3 strength; Gene = if the sample contained a pathogenic mutation or rare variant this column specifies the gene that mutation/variant was identified in; GermlineVariant = CSN annotation for the identified pathogenic mutation or rare variant assessed for that sample; GermlineVariantGroup = 'Pathogenic Mutation' are samples in which a germline BRCA pathogenic mutation was identified, 'RareVariant' are samples that did not have a BRCA pathogenic mutation but did have a variant present at <0.1% variant frequency in the ExAC v0.3 non-TCGA, 'Baseline' are samples without a BRCA pathogenic mutation or rare variant but were heterozygous for a common nonsynonymous variant in BRCA (see methods), or '.' the sample does not belong to any of the groups.

Table S2. Summary of 813 BRCA variants identified in the 7632 individuals with cancer

Description of column headings are given below:

Gene = Gene symbol; Variant = CSN annotation of the variant; Class = CAVA v1.2 class of the variant (ESS = essential splice site, FS = frame-shift, IF = in-frame indel, NSY = nonsynonymous, SG = stop-gain, SS = splice site, and SY = synonymous); TCGA_VarCount = number of samples in which the variant was called in the 7632 TCGA samples; BROV_VarCount = number of samples with breast or ovarian cancer in the TCGA in which the variant was called; OtherCancer_VarCount = TCGA_VarCount – BROV_VarCount; ExAC_VarCount = number of samples containing the variant in the ExAC v0.3 non-TCGA data; ClinVarInterpretation = the most common variant interpretation or severe (in case of ties) from ClinVar on 13/03/2018; GermlineVariantGroup = the group in which the variant belongs to (see methods).

Table S3. Number of samples of the 28 cancer types investigated and the number with germline BRCA pathogenic mutations

Table S4. Summary of BRCA variants identified in 1000 UK population controls

Description of column headings are the same as for Supplementary Table 2.

Table S5. Summary of germline and somatic BRCA data in 187 breast/ovarian cancers with Signature 3

Description of column headings are given below:

ID = TCGA Barcode; Cancer = abbreviation of the primary cancer diagnosis; AgeAtDx = Age at diagnosis; Gender = sample's gender; Gene = if the sample contained a pathogenic mutation or rare variant this column specifies the gene that mutation/variant was identified in; GermlineVariant = CSN annotation for the identified pathogenic mutation or rare variant assessed for that sample; GermlineVariantGroup = 'Pathogenic Mutation' are samples in which a germline BRCA pathogenic mutation was identified, 'RareVariant' are samples that did not have a BRCA pathogenic mutation but did have a variant present at <0.1% variant frequency in the ExAC v0.3 non-TCGA, 'Baseline' are samples without a BRCA pathogenic mutation or rare variant but were heterozygous for a common nonsynonymous variant in BRCA (see methods), or '.' the sample does not belong to any of the groups; Somatic BRCA eventsx2 = comma separated list of somatic events that were identified in the sample if that sample contained at least 2 somatic events.
